# Development and validation of a predictive model based upon extracellular vesicle-derived transposable elements for non-invasive detection of pancreatic adenocarcinoma

**DOI:** 10.1186/s40364-025-00770-6

**Published:** 2025-04-05

**Authors:** Yueting Liang, Xin Sui, Shuai Li, Haoxin Peng, Wenyi Jiang, Minqi Jia, Shaoran Jiang, Weihu Wang, Huajing Teng

**Affiliations:** 1 Department of Radiation Oncology, Key Laboratory of Carcinogenesis and Translational Research (Ministry of Education/Beijing), Peking University Cancer Hospital & Institute, Beijing, 100142 China; 2 Department of Gastrointestinal Oncology, Key Laboratory of Carcinogenesis and Translational Research (Ministry of Education/Beijing), Peking University Cancer Hospital & Institute, Beijing, 100142 China

**Keywords:** Diagnostic biomarker, Transposable element, Pancreatic adenocarcinoma, Extracellular vesicle

## Abstract

Pancreatic adenocarcinoma (PAAD) is a highly lethal malignancy that leads to patients missing optimal treatment opportunities due to its atypical clinical symptoms and the lack of effective diagnostic biomarkers. To develop a biomarker panel based on extracellular vesicle-derived transposable elements (EV-TEs) for non-invasive detection of PAAD, we analyzed 6.75 Tbp sequencing data of 852 EV-derived transcriptomes from two cohorts, and identified 31 EV-TEs features as the biomarker panel using recursive feature elimination. Predictive model constructed using the Support Vector Machine (SVM) algorithm demonstrated excellent performance for PAAD detection in the training set (AUC: 0.90, 95% CI: 0.86–0.93), the test set (AUC: 0.86, 95% CI: 0.79–0.92) and the independent external validation cohort of blood EV-derived samples (AUC: 0.88, 95% CI: 0.84–0.92). This study presents the first EV-TEs based predictive model for PAAD detection, showcasing the immense potential of these ‘junk DNA’ as innovative diagnostic biomarker for cancers.

**Supplementary Information:**

The online version contains supplementary material available at 10.1186/s40364-025-00770-6.

**To the editor,**

Pancreatic adenocarcinoma (PAAD) accounts for approximately 5% of all cancer deaths worldwide [1], and about 90% of PAAD patients are diagnosed at advanced stages with 5-year survival below 13% [2]. Currently, carbohydrate antigen 19-9 (CA19-9) is widely used as an auxiliary diagnostic biomarker for PAAD but exhibits limited specificity and sensitivity, underscoring the pressing need to search for novel biomarkers for early diagnosis of PAAD [3].

Extracellular vesicles (EVs) can transmit biomolecules from parent cells and exhibit strong stability in bodily fluids [4]. Their unique characteristics herald a promising non-invasive approach for early cancer detection [5, 6]. Transposable elements (TEs), once considered ‘junk DNA’, are now recognized as pivotal player in cancer development, and they are enriched in cell-free transcriptomes and preferentially packaged in cancer-derived EVs [7]. However, the potential of EV-derived TEs (EV-TEs) as non-invasive biomarkers for PAAD diagnosis remains unexamined. Here, we analyzed 6.75 terabases of transcriptome sequencing data and employed three machine learning algorithms to construct PAAD predictive models based on EV-TEs.

A total of 284 PAAD patients, 100 patients with chronic pancreatitis (CP) and 117 healthy controls were included in the discovery cohort [8]. Meanwhile, a dataset including 150 PAAD, 49 CP and 152 healthy samples was designated as the external validation cohort (Table S1) [9]. Altogether, 852 EV-derived transcriptomes were processed, and quantification for each TE were obtained (Fig. 1A-C). To identify the optimal set of EV-TE features for predicting PAAD, we employed the recursive feature elimination method in the discovery cohort, and ultimately selected 31 features as our EV-TEs biomarker panel (Fig.S1A). Of the features, some were reported to be activated in cancers. For example, HERV1_I − int has been significantly activated in colon adenocarcinoma [10], and upregulated expression of LTR48B have been reported in multiple tumor types [10]. A correlation analysis revealed that most of the 31 features exhibited weak to no correlation, indicating their independent contributions to the model (Fig.S1B). Principal component analysis based on the biomarker panel effectively distinguishes PAAD from CP and healthy individuals (Fig. 1D). We then evaluated the predictive performance of the EV-TEs biomarker panel using three algorithms. Initially, we randomly split the discovery cohort into training (70%) and test (30%) sets. We then conducted a comparative analysis of clinical characteristics between PAAD and control (CP patients and healthy individuals) groups in different set (Table S2-S5). Among the algorithms, the Support Vector Machine (SVM) model excelled, achieving an average area under the curve (AUC) of 0.90 and a Kappa coefficient of 0.66 in the training cohort (Figs. 1E-F and 2A; Table S6). In the test set, the SVM model also demonstrated promising performance, with an average AUC of 0.86 (Fig. 2D; Table S7). Binary classifications were then conducted leveraging its prediction strength, and the result further substantiated the SVM model’s robust ability to distinguish PAAD from CP and healthy controls (Fig. 2B, E,H). The training set confusion matrix revealed that 32 of 39 PAAD patients (82.1%) were accurately classified as true positives, and 30 of 31 healthy individuals (96.8%) were correctly identified as true negatives (Fig. 2C). Similarly, in the test set, the model successfully classified 70 of 85 PAAD patients (82.4%) as true positives and accurately identified 55 of 66 healthy individuals (83.3%) as true negatives (Fig. 2F). These findings underscore the potential of SVM modeling for diagnosis in PAAD.

**Fig. 1:**
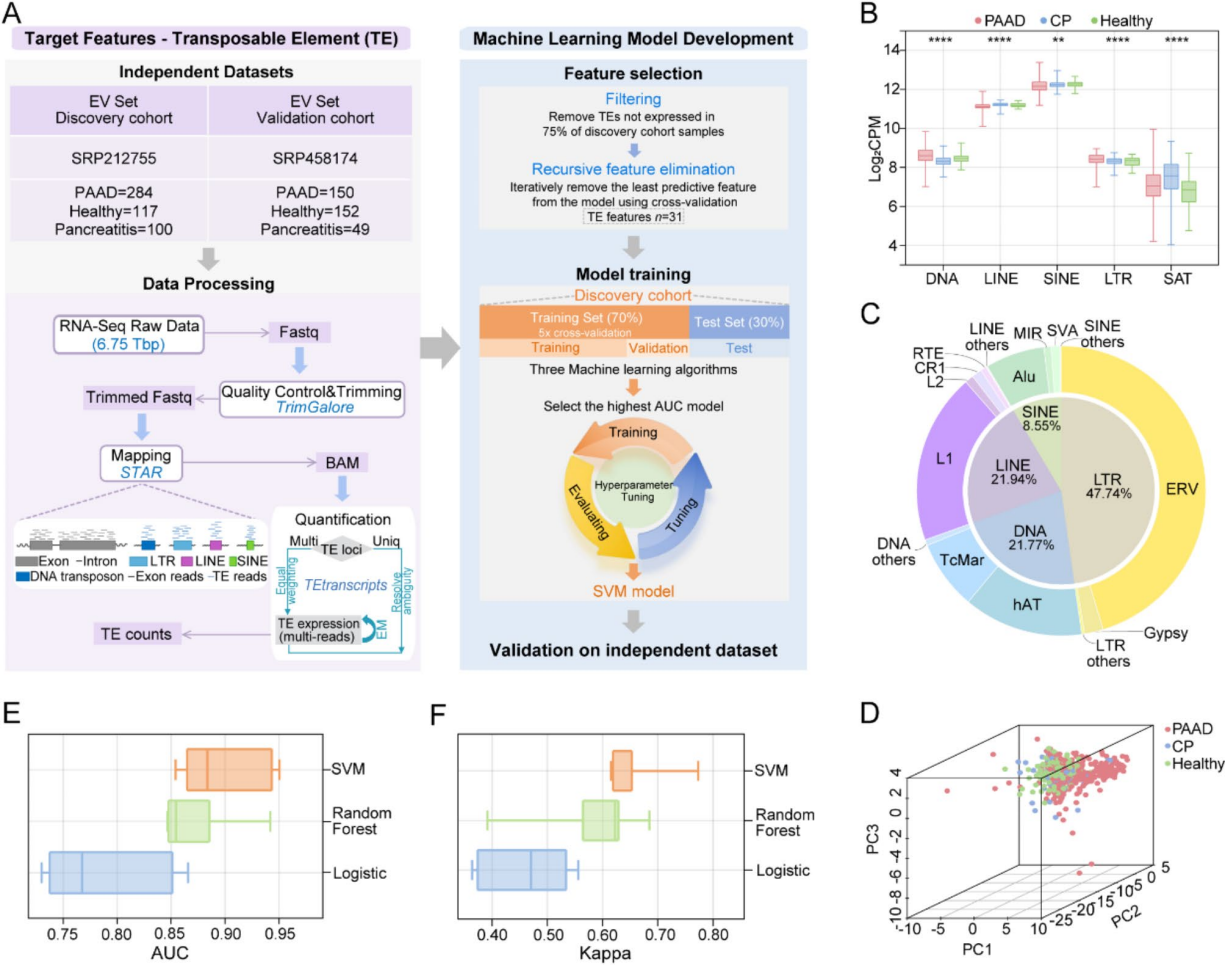
The profile of plasma extracellular vesicle-derived transposable elements (EV-TEs) differs between PAAD, CP patients and healthy individuals. **A** Workflow of the study design for the identification of a plasma EV-derived TE panel as a biomarker for the diagnosis of PAAD patients. **B** Expression level of different types of repetitive sequences in PAAD patients versus CP and healthy control samples. **C** Given the lack of expression of certain TEs in the majority of samples, we performed filtering, and the remaining TEs (620) types exhibited a wide range of diversity. **D** Principal component analysis of PAAD, CP patients and healthy individuals based on the expression of 31 candidate EV-TEs. **E** Area under curve (AUC) performance for each machine learning model based on EV-TEs biomarker panel. **F** Kappa consistency value for each machine learning model based on the EV-TEs biomarker panel. PAAD, pancreatic adenocarcinoma; CP, chronic pancreatitis; DNA, DNA transposons; LINE, long interspersed nuclear elements; LTR, long terminal repeat; SINE, short interspersed nuclear elements; SAT, satellite sequence; CPM, counts per million; SVM, support vector machine. EM, Expectation-maximization algorithm

**Fig. 2:**
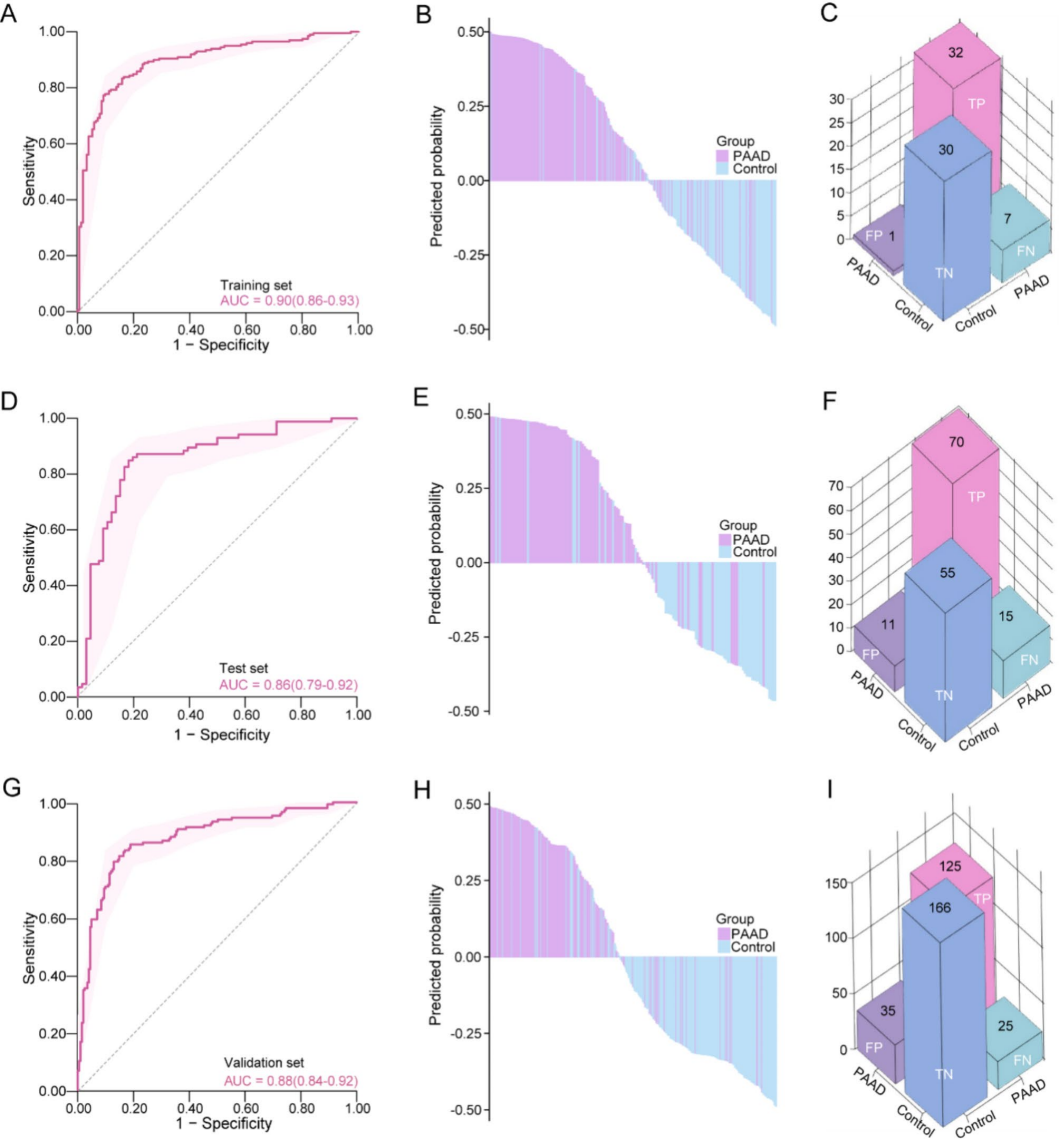
The diagnostic efficiency of plasma extracellular vesicle-derived transposable elements (EV-TEs) biomarker panel in detecting PAAD. **A-I** Receiver operating characteristic curve (ROC), binary classifications waterfall plot, and confusion matrix for EV-TEs panel for PAAD diagnosis in the training, test and external validation set. PAAD, pancreatic adenocarcinoma; TP, true-positive; FP, false-positive; TN, true-negative; FN, false-negative

To verify the reliability of the model, we conducted an external validation using an independent cohort. ROC analysis robustly demonstrated that it could distinguish PAAD from CP and healthy controls with an AUC value of 0.88 (Fig. 2G). Consistent with these results, the confusion matrix for the validation set also showed that the model successfully classified 125 of 150 PAAD patients (83.3%) as true positives and accurately identified 166 of 201 healthy individuals (82.6%) as true negatives (Fig. 2I). CA19-9, the clinically widely-used diagnostic biomarker for PAAD, exhibits limited sensitivity (73.4–77.4%) and specificity (75.4–79.7%) [3, 11]. The findings above strengthened the potential of our EV-TEs panel as a non-invasive diagnostic tool for PAAD detection (Table S7), although we haven’t compared its performance with that based on CA19-9 of the same cohort.

Despite its achievements, the study faces key challenges. Age distributions differed between groups, with the PAAD group being older than the control group. Although this mirrors clinical observations [12], we cannot exclude the confounding effect of age on the performance of our established prediction model. Furthermore, the absence of staging data for each patient hampers the interpretation of the biological significance of our panel in the detection of early stage PAAD patients. Thus, further validation in large-scale, multicenter trials is needed to fully evaluate the performance of the panel for early diagnosis of PAAD.

## Electronic supplementary material

Below is the link to the electronic supplementary material.

Supplementary Material 1

## Data Availability

All data and materials can be available from the corresponding authors, who can be contacted on the email address.

## Declarations

### Abbreviations

PAAD Pancreatic adenocarcinoma.

CP Chronic pancreatitis.

CA 19-9 Carbohydrate antigen 19-9.

EV-TEs Extracellular vesicle-derived transposable elements.

EVs Extracellular vesicles.

TEs Transposable elements.

SATs Satellite sequences.

LTRs Long terminal repeats.

LINEs Long interspersed nuclear elements.

SINEs Short interspersed nuclear elements.

AUC Area under the curve.

LR Logistic Regression.

RF Random Forest.

SVM Support Vector Machine.
