# Supplementary material for: Development and validation of a predictive model based upon extracellular vesicle-derived transposable elements for non-invasive detection of pancreatic adenocarcinoma
Source: Biomark Res. 2025 Apr 5;13:54. doi: 10.1186/s40364-025-00770-6 (PMC11972517; doi:10.1186/s40364-025-00770-6)
Supplement: Supplementary file 1 — Supplementary Material 1 [file 40364_2025_770_MOESM1_ESM.docx]

**Supplementary Files for**

**Development and validation of a predictive model based upon** **extracellular vesicle-derived transposable elements for non-invasive detection of pancreatic adenocarcinoma**

**Materials and Method**

**Data collection and processing**

The extracellular vesicles-derived transcriptome sequences from two independent pancreas-related datasets, including SRP212755^1^ and SRP458174^2^, were obtained from the Sequence Read Archive of National Center for Biotechnology Information. The methods for purifying EVs and RNA-seq library preparation were consistent in these two datasets, both of which used the exoRNeasy Serum/Plasma kit (Qiagen, Hilden, Germany) to isolate EVs by affinity-based binding, and both of which used SMARTer Stranded Total RNA-Seq Kit - Pico Input Mammalian (Clontech, Palo Alto, California, USA) to construct RNA-seq library. Specifically, the SRP212755 dataset comprises 284 samples of pancreatic adenocarcinoma (PAAD), including 59 cases in stage I, 77 in stage II, 42 in stage III, and 106 in stage IV. Furthermore, the dataset includes 100 samples of chronic pancreatitis (CP) and 117 healthy control samples. Of the SRP212755 dataset participants, 323 were recruited from Fudan University Shanghai Cancer Center, while 178 participants were recruited from Changhai Hospital of the Second Military Medical University (Shanghai, China) and Xi’an Jiaotong University Affiliated Medical Center (Xi’an, China)^1^. Additionally, blood samples for the SRP212755 dataset were collected from all participants in 10 mL EDTA-coated Vacutainer tubes^1^. Blood samples of PAAD patients were collected before surgery for those with resectable tumors and before chemotherapy for those with unresectable tumors^1^. Five patients received neoadjuvant chemotherapy (regimens included gemcitabine/nab-paclitaxel or gemcitabine/oxaliplatin), and their blood samples were collected before the start of neoadjuvant chemotherapy^1^. Meanwhile, the SRP458174 dataset includes a total of 150 PAAD, 49 CP and 152 healthy samples. However, the detailed information regarding the sample collection for this cohort has not yet been obtained. The lack of this information may introduce potential confounding factors, which could affect the comparison of the two datasets and might limit the interpretation of the results. Clinical features for those datasets are recorded in Table S1. For diagnostic model development and validation, we designated the samples from SRP212755 as the discovery cohort, the SRP458174 dataset as validation cohort. The discovery cohort were randomly divided into two independent subsets with different proportions, 70% of the randomly selected samples were used for training and the remaining 30% for testing. Comparative analysis of clinical characteristics between PAAD and control (CP patients and healthy individuals) groups in training, test, and external validation sets is recorded in Table S2-S5.

We performed RNA-seq analysis on the above samples. Adapter sequences and low quality reads from raw RNA sequencing data were trimmed by TrimGalore v0.4.5. Trimmed sequencing reads were aligned to the reference genome sequence (GRCh38/hg38) using the STAR aligned v2.7.11a, allowing a certain degree of multimapping^3^. For read counting per annotated TE, we have employed the TEtranscripts v.2.2.3 to tally reads aligning to TEs in the hg38 RepeatMasker annotation^4^. This process was based on BAM files derived from the aligned and sorted reads of each individual sample, and expectation-maximization algorithm was used to determine multi-reads assignments to all TE transcripts^4^ (Fig. 1A). Further data processing was performed with the R package edgeR v.4.0.16, which resulted in the generation of counts-per-million (CPM) values for each TE^5^.

**Feature Selection**

To identify key TE features for PAAD prediction, we first filtered out TEs that were not expressed in at least 75% of the discovery cohort samples, as a means to mitigate data noise, leaving us with 620 TE features. Then, in this cohort, we employed Recursive Feature Elimination (RFE), a feature selection method that iteratively removes the least important features, based on the importance ranking provided by a base estimator, until the desired number of features is reached. For our study, we used RFE based on the Random Forest algorithm to further refine the set of significant TEs, enhancing stability through 5-fold cross-validation. Additionally, we considered feature subsets ranging from 1 to 50 to facilitate the exploration of the optimal feature set for our predictive model. Through this rigorous process, we ultimately identified 31 EV-TE features that exhibited the highest predictive power for PAAD.

**Dimensionality reduction and visualization**

The visualization of PAAD, CP and healthy data was achieved through the low-dimensional embedding technique of Principal Component Analysis (PCA), where EV-TEs were considered as the key features. PCA cluster analysis was conducted using the R packages scatterplot3d (version 0.3.44) under default settings.

**Construction of predictive models based on three machine learning methods**

The machine learning models employed in our study, specifically Random Forest (RF), Support Vector Machine (SVM) and Logistic Regression (LR), were trained to differentiate between PAAD and Control (CP and healthy individuals) samples. SVM, a classification and regression analysis algorithm, identifies the optimal hyperplane for distinguishing between two data classes^6^. It accomplishes this by potentially mapping sample vectors into a higher-dimensional space and finding an optimal hyperplane to maximize the margin between the two classes^6^. RF leverages multiple decision trees, each trained on distinct subsets of the training data, to minimize variance through prediction averaging. LR is a statistical method for binary classification problems, and can be viewed as a special case of generalized linear models (GLM).

The discovery cohort was initially split into a 70% subset for model training and a 30% subset reserved as a test set for final model evaluation. Following the feature selection process described above, 31 EV-TEs were chosen for integration into the model during its training phase. To optimize the hyperparameters for our machine learning models, we employed 5-fold cross-validation. Specifically, we randomly shuffled the training subset and divided it into five folds. In each iteration, ranging from 1 to 5, the i^th^ fold was designated as the validation set, while the remaining folds collectively served as the training data. The optimal hyperparameters were determined by averaging performance across validation sets. We comparatively evaluated the diagnostic performance of the optimal models from various machine learning algorithms, primarily using the Area Under the receiver operating characteristic (ROC) Curve (AUC) as our evaluation metric. Additionally, we employed other evaluation metrics such as accuracy, sensitivity, specificity, precision, and F1-score to provide a comprehensive assessment.

Among the three machine learning algorithms tested, the SVM exhibited the highest AUC. The optimal hyperparameters for the SVM, determined through cross-validation, were {'cost': 2.2, 'gamma': 0.023}. Utilizing these parameters, we trained our SVM model, incorporating a Radial Basis Function (RBF) kernel.

**Validating and Evaluating the Diagnostic Performance of SVM** **Predictive Models in Independent Validation Sets**

To validate and evaluate the diagnostic performance of SVM predictive models (PAAD versus Control (CP and healthy individuals)) built upon the 31 selected EV-TE features, we employed an external independent validation set, with all SVM parameters fixed. The diagnostic performance of the SVM predictive models was evaluated by AUC.

**Statistical Analysis**

All data processing and statistical analysis were conducted within the R statistical software environment (version 4.3.1). Heatmaps visualizing differentially expressed transposable elements among PAAD, CP and healthy groups were generated using the ComplexHeatmap package (version 2.13.1). For machine learning approaches, SVM were implemented via the 'e1071' package (version 1.7.14), RF were applied using the 'randomForest' package (version 4.7.1.1), and LR analyses were performed with the 'stats' package (version 4.3.1). The ROC curves and the AUC values were computed to assess the performance of the diagnostic biomarkers, employing the “pROC” package (version 1.18.5). Accuracy, Kappa, sensitivity, specificity, precision, F1-score, positive predictive value (PPV) and negative predictive value (NPV) were calculated by utilizing the ‘confusionMatrix’ function from the R package ‘caret’ (version 6.0.94). When appropriate, continuous variables were compared among different groups using the Kruskal-Wallis test. A p-value less than 0.05 was considered statistically significant.

**
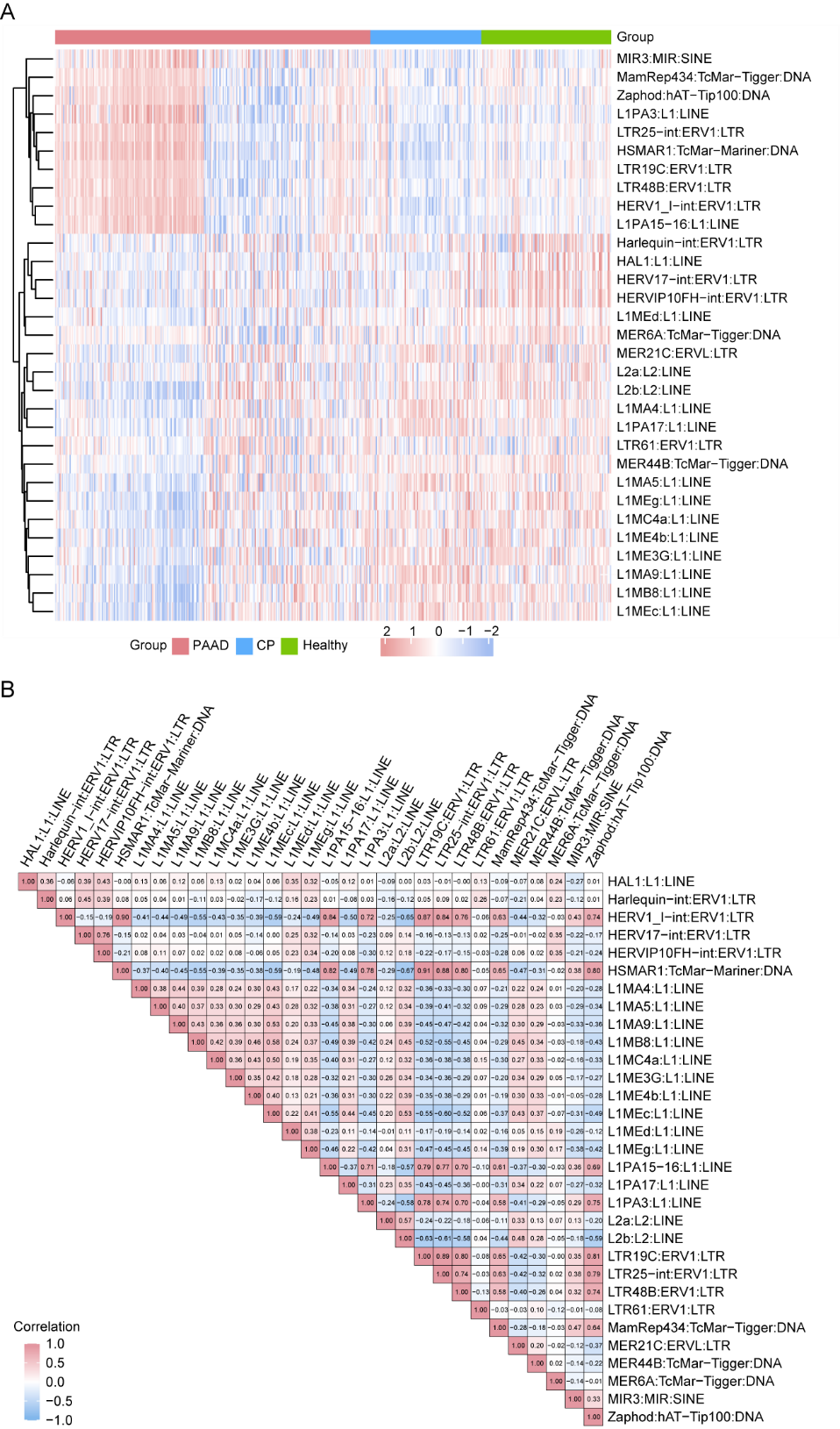
**

**Supplementary Fig. S1 A** Heatmap of hierarchical clustering showing the 31 EV-TEs among PAAD, CP and healthy controls in the discovery cohort. Each row represents a candidate EV-TE, and each column represents an individual sample. The scale represents the expression values. **B** Correlation matrix of 31 candidates EV-TEs.

**Supplementary Table S1.** Characteristics of patients for the discovery and external validation cohorts

|  |  | Discovery cohort (*n*=501) | | | Validation cohort (*n*=351) | | |
| --- | --- | --- | --- | --- | --- | --- | --- |
|  |  | SRP212755 | | | SRP458172 | | |
|  |  | PAAD  (*n*=284) | CP  (*n*=100) | Healthy  (*n*=117) | PAAD  (*n*=150) | CP  (*n*=49) | Healthy  (*n*=152) |
| Sex, *n* | Male  Female | 167  117 | NA | 71  46 | 94  56 | 32  17 | 83  69 |
| Age, years | Median  Range | 62  31-84 | NA | 60  41-91 | 61  35-83 | 46  11-76 | 55  27-91 |
| Serum CA19-9, U/mL | Median  Range | 516.9 | NA | NA | NA | NA | NA |
|  |  | 0.6-1200 |  |  |  |  |  |
| PAAD Stage, *n* | I  II  III  IV | 59  77  42 | - | - | NA | - | - |
|  |  | 106 |  |  |  |  |  |

Abbreviations: PAAD, Pancreatic adenocarcinoma; CP, Chronic pancreatitis; CA19-9, Carbohydrate antigen 19-9; NA, Not available.

**Supplementary Table S2.** Clinical characteristics between PAAD and control groups within training, test, and external validation sets

| Characteristics | Training set (*n*=349) | Test set (*n*=152) | External validation set (*n*=351) | *P* value |
| --- | --- | --- | --- | --- |
| Disease state, *n* |  |  |  | < 0.001 |
| PAAD | 198 | 86 | 150 |  |
| Control (CP+Healthy) | 151 | 66 | 201 |  |
| Sex, *n* |  |  |  | 0.935 |
| Male | 169 | 69 | 209 |  |
| Female | 113 | 50 | 142 |  |
| Age, years, median (IQR) | 62 (54, 68) | 61 (54, 67.5) | 58 (49, 66) | < 0.001 |
| CA19-9, mean ± sd | 540.66 ± 456.52 | 550.58 ± 448.18 | NA |  |

Abbreviations: PAAD, Pancreatic adenocarcinoma; CP, Chronic pancreatitis; CA19-9, Carbohydrate antigen 19-9; NA, Not available.

*The clinical information for CP in the training and test sets is not available.

**Supplementary Table S3.** Comparative analysis of clinical characteristics between PAAD and control groups in the training set

| Characteristics | PAAD (*n*=198) | Control (CP+Healthy) (*n*=151) | *P* value |
| --- | --- | --- | --- |
| Sex, *n* |  |  | 0.861 |
| Male | 118 | 51 |  |
| Female | 80 | 33 |  |
| Age, years, median (IQR) | 62 (56, 67) | 61 (51.75, 68.25) | 0.200 |
| CA19-9, mean ± sd | 540.66 ± 456.52 | NA |  |

Abbreviations: PAAD, Pancreatic adenocarcinoma; CP, Chronic pancreatitis; CA19-9, Carbohydrate antigen 19-9; NA, Not available.

*The clinical information for CP in the training set is not available.

**Supplementary Table S4.** Comparative analysis of clinical characteristics between PAAD and control groups in the test set

| Characteristics | PAAD (*n*=86) | Control (CP+Healthy) (*n*=66) | *P* value |
| --- | --- | --- | --- |
| Sex, *n* |  |  | 0.72 |
| Male | 49 | 20 |  |
| Female | 37 | 13 |  |
| Age, years, median (IQR) | 62.5 (57, 68) | 57 (52, 64) | 0.018 |
| CA19-9, mean ± sd | 550.58 ± 448.18 | NA |  |

Abbreviations: PAAD, Pancreatic adenocarcinoma; CP, Chronic pancreatitis; CA19-9, Carbohydrate antigen 19-9; NA, Not available.

*The clinical information for CP in the test set is not available.

**Supplementary Table S5.** Comparative analysis of clinical characteristics between PAAD and control groups in the external validation set

| Characteristics | PAAD (*n*=150) | Control (CP+Healthy) (*n*=201) | *P* value |
| --- | --- | --- | --- |
| Sex, *n* |  |  | 0.303 |
| Male | 94 | 115 |  |
| Female | 56 | 86 |  |
| Age, years, mean ± sd | 61.047 ± 9.191 | 52.761 ± 14.773 | <0.001 |
| CA19-9, mean ± sd | NA | NA |  |

Abbreviations: PAAD, Pancreatic adenocarcinoma; CP, Chronic pancreatitis; CA19-9, Carbohydrate antigen 19-9; NA, Not available.

**Supplementary Table S6.** AUC and Kappa values for each fold of 5-fold cross-validation in training set based on the EV-TEs biomarker panel

|  | SVM | | RF | | Logistic | |
| --- | --- | --- | --- | --- | --- | --- |
|  | AUC | Kappa | AUC | Kappa | AUC | Kappa |
| fold_1 | 0.9433333 | 0.6529 | 0.8854167 | 0.6286 | 0.73 | 0.374 |
| fold_2 | 0.865 | 0.6192 | 0.8475 | 0.5643 | 0.7375 | 0.4706 |
| fold_3 | 0.8541667 | 0.6192 | 0.8545833 | 0.6224 | 0.7675 | 0.3636 |
| fold_4 | 0.8837607 | 0.6152 | 0.8465812 | 0.3915 | 0.865812 | 0.556 |
| fold_5 | 0.9503722 | 0.7729 | 0.9421009 | 0.6847 | 0.8511166 | 0.5337 |

Abbreviations: AUC, Area under the curve; SVM, Support Vector Machine; RF, Random Forest.

**Supplementary Table S7.** Performance of the SVM model in training, test, and validation sets

|  | Training set | Test set | Validation set |
| --- | --- | --- | --- |
| AUC (95% CI) | 0.90 (0.86, 0.93) | 0.86(0.79,0.92) | 0.88 (0.84, 0.92) |
| Accuracy | 0.89 | 0.82 | 0.83 |
| TP | 32 | 70 | 125 |
| FP | 1 | 11 | 35 |
| FN | 7 | 16 | 25 |
| TN | 30 | 55 | 166 |
| Precision | 0.97 | 0.86 | 0.78 |
| F1-score | 0.89 | 0.84 | 0.81 |
| Sensitivity | 0.82 | 0.81 | 0.83 |
| Specificity | 0.97 | 0.83 | 0.83 |

Abbreviations: AUC, Area under the curve; SVM, Support Vector Machine; TP, True-positive; FP, False-positive; FP, False-negative; TN, True-negative.

**References**

1. Yu S, Li Y, Liao Z, et al. Plasma extracellular vesicle long RNA profiling identifies a diagnostic signature for the detection of pancreatic ductal adenocarcinoma. *Gut*. 2020;69(3):540-550.
2. Zhao J, Li Q, Hu J, et al. Circular RNA landscape in extracellular vesicles from human biofluids. *Genome Medicine*. 2024;16(1):126.
3. Dobin A, Davis CA, Schlesinger F, et al. STAR: ultrafast universal RNA-seq aligner. *Bioinformatics (Oxford, England)*. 2013;29(1):15-21.
4. Jin Y, Tam OH, Paniagua E, Hammell M. TEtranscripts: a package for including transposable elements in differential expression analysis of RNA-seq datasets. *Bioinformatics (Oxford, England)*. 2015;31(22):3593-3599.
5. Robinson MD, McCarthy DJ, Smyth GK. edgeR: a Bioconductor package for differential expression analysis of digital gene expression data. *Bioinformatics (Oxford, England)*. 2010;26(1):139-140.
6. Smola AJ, Schölkopf B. A tutorial on support vector regression. *Statist Comput*. 2004;14(3):199-222.
